# Investigating Different Duplication Pattern of Essential Genes in Mouse and Human

**DOI:** 10.1371/journal.pone.0120784

**Published:** 2015-03-09

**Authors:** Debarun Acharya, Dola Mukherjee, Soumita Podder, Tapash C. Ghosh

**Affiliations:** Bioinformatics Centre, Bose Institute, Kolkata, West Bengal, India

## Abstract

Gene duplication is one of the major driving forces shaping genome and organism evolution and thought to be itself regulated by some intrinsic properties of the gene. Comparing the essential genes among mouse and human, we observed that the essential genes avoid duplication in mouse while prefer to remain duplicated in humans. In this study, we wanted to explore the reasons behind such differences in gene essentiality by cross-species comparison of human and mouse. Moreover, we examined essential genes that are duplicated in humans are functionally more redundant than that in mouse. The proportion of paralog pseudogenization of essential genes is higher in mouse than that of humans. These duplicates of essential genes are under stringent dosage regulation in human than in mouse. We also observed slower evolutionary rate in the paralogs of human essential genes than the mouse counterpart. Together, these results clearly indicate that human essential genes are retained as duplicates to serve as backed up copies that may shield themselves from harmful mutations.

## Introduction

Gene duplication was thought to be one of the major driving factors stimulating genome and organism evolution [1–4], as it provides raw genetic materials for structural and functional modification and at the same time conserves the parental function. Although, gene duplication is not always beneficial, and most duplicates become subsequently inactivated or pseudogenized in the genome [4], it may have many implications in an organism’s life. For example, the duplicates may be maintained in the genome for its immediate benefit to the organism, like increased gene dosage [5] or serve as backup copies to restore the function if the original one becomes deleted [6,7]. Apart from this, the duplicates may undergo modifications to take up novel functions, i.e. neofunctionalization [4], or they may share their function after complementary degenerative mutations, i.e. subfunctionalization [8,9]. The pattern of gene duplication may vary between species and also across different groups of genes within the same species. Several factors contributing gene duplication has been observed till date in diverse organisms like protein connectivity and protein interaction network [10–12], protein complexity [13,14], gene retention and sequence divergence [15], dosage balance [16] and nevertheless, gene essentiality [17–19].

Essential genes are indispensable to an organism and cause severe reduction in its fitness like sterility or lethality upon deletion [20]. These genes are mainly associated with important biological functions. However, many expressed genes performing such functions are considered to be nonessential, as their deletion can be compensated by other genes having similar or identical functions and expression [21]. Gene duplication is an important mechanism for such functional redundancy to occur [4]. Now, there may be two kinds of possibilities for essential genes to prefer or avoid the course of gene duplication. First, essential genes are required to become duplicated for providing backup copies that could shield themselves from any harmful mutations; secondly from evolutionary standpoint, essential genes may prefer to stay away from gene duplication since ectopic recombination and replication driven gene duplication may increase the chances of mutational load which is not at all acceptable for essential genes for being the most conserved gene-group [22,23].

Gene essentiality was widely studied across model organisms and shown to bear a complex relationship with gene duplication [19]. In lower eukaryotes like yeast, a higher proportion of essential genes were observed in singletons than in duplicates [7]. However, studies with mouse showed that the proportion of essential genes in duplicates are comparable to that in singletons [10,18]. Additionally, two follow-up studies with mouse also report that the proportion of essential genes is higher in singletons than in duplicates [21,24].

Till date, all the studies regarding essential genes were carried in yeast and mouse due to unavailability of human gene essentiality data. In a previous study, researchers attempted to explore the properties of human orthologs of mouse essential genes [25]. However, considering such human orthologs as essential may not be accurate [26]. Taking advantage of the Online Gene Essentiality (OGEE) database that represents a valuable resource of human and mouse essential genes, we performed a comprehensive analysis comparing duplication pattern of essential genes in human and mouse. We noticed that in mouse, the essential genes prefer to remain as singleton whereas the trend is reverse for human, which is unexplored so far. We have also explored the underlying reasons and the benefits of maintaining essential genes as duplicates in humans.

## Materials and Methods

### Gene Essentiality and Gene Duplication

Gene essentiality and duplication of human (*Homo sapiens*) and mouse (*Mus musculus*) were obtained from the Online Gene Essentiality (OGEE) database (http://ogeedb.embl.de) [27] (S1 Dataset). The paralog lists for human and mouse essential genes were provided by the authors of OGEE database [27] (S2 Dataset).

### Developmental Genes

The developmental genes for mouse and human were obtained from Online Gene Essentiality (OGEE) database [27] (S1 Dataset). Here, a gene is considered as developmental if they are associated with one of the two GO terms: GO:0007275 (multicellular organismal development) and GO:0030154 (cell differentiation) or their daughter terms, and others as non-developmental, a method adapted by Makino et al. 2009 [19].

### Phyletic Age and Overall Proportion of Essentiality

Phyletic origin of a gene can be defined as the most distance group of organisms where the homologs (orthologs) of that gene are present. The phyletic age of human and mouse genes was obtained from the Online Gene Essentiality (OGEE) database [27], where the authors used the phyletic age prediction algorithm described by Wolf et al. [28]. The genes were divided in seven classes according to their evolutionary origin, namely 0 (not assigned), 1 (Mammalia), 2 (Chordata), 3 (Metazoa), 4 (Fungi/Metazoa group), 5 (Eukaryota) and 6 (cellular organisms). We discarded the first group in which the phyletic age was not assigned and selected the rest from mouse and human OGEE genes. We obtained the final mouse and human data with gene essentiality, gene duplication and phyletic age information containing 5869 and 18400 genes, respectively. We divided the human and mouse OGEE genes into two groups depending on their phyletic age: the ‘old duplicates’ (containing three older classes) and ‘new duplicates’ (containing the rest three classes) in both human and mouse (S1 Dataset). From this data, we calculated the overall proportion of essential genes in singletons and duplicates for both species as a weighted average using this formula [21]:

$$P_{E}=\;f_{old}\times P_{E}^{old}+f_{young}\times P_{E}^{young}$$

Where, *f*_*old*_ and *f*_*young*_ are the fraction of old and young genes contained in the gene group and the $P_{E}^{old}$ and $P_{E}^{young}$ are proportion of essential genes in old and young counterparts. Using this formula, we calculated the proportion of essential genes in singleton and duplicates for both species irrespective of their age bias.

### Functional Distance

The functional distance for the human and mouse essential genes carried by the Gene Ontology (GO) annotations was calculated using the GO domain molecular function for essential genes and their paralogous copies of corresponding species from Ensembl 71 biomart interface (http://www.ensembl.org/biomart/martview) [29]. The GO terms for each human and mouse essential gene and the corresponding paralogous genes were calculated separately. Using the Czekanowski—Dice distance formula [30] mentioned below, we calculated the functional divergence for each human and mouse essential genes with their paralogous counterparts.

$$Functional\;distance\;(i,j)=\;\frac{Number\;of\;Terms(i)\Delta Terms(j)}{\left[Number\;of\;(Terms(i)\cup Terms(j))+Number\;of(Terms(i)\cap Terms(j))\right]}$$

In which, i and j denote a gene and its paralogous gene within a species. Terms (i) and Terms (j) are the lists of the GO terms for individual genes. ‘∪’ and ‘∩’ denotes the nonredundant and common GO id count, respectively, of the two genes. ‘Δ’ is the symmetrical difference between the GO term sets of two genes, i.e. ‘(∪−∩)’.

Although the Czekanowski-Dice distance formula is the most commonly used method for calculation of functional distance, it is sensitive to the number of GO terms per gene and therefore may be erroneous for cross-species comparison. Therefore, to compare the functional distance between mouse and human essential genes using the Czekanowski-Dice formula, we must consider the number of GO terms associated with the genes. To ensure that, we binned our functional distance data of the two species in three groups: Group A (with GO terms 1 to 4; N_human_ = 367, N_mouse_ = 773), Group B (with GO terms 5 to 8; N_human_ = 343, N_mouse_ = 485) and Group C (with GO terms > 8; N_human_ = 244, N_mouse_ = 278) and compared the functional distance of human and mouse essential genes within each group.

### Pseudogenization

Mouse and human pseudogenes were obtained from the biomart interface of ensemble 71 (http://www.ensembl.org/biomart/martview) [29]. For both the species, we searched for the gene IDs for which the gene biotype contains the term ‘pseudogene’. This includes pseudogene, IG-V-pseudogene, TR-V-pseudogene, polymorphic pseudogene, TR-J-pseudogene, IG-C-pseudogene, IG-J-pseudogene and processed pseudogene. We calculated the proportion of paralog pseudogenization by considering only the duplicated essential genes with at least one pseudogenized paralog. The proportion of paralog pseudogenization was calculated by the ratio of the number of pseudogenized paralogs and the total number of paralogs. The mouse and human essential genes with the biotype of the paralog are provided in S3 Dataset.

### Micro-RNA Target Sites

Average micro-RNA target sites for human and mouse were obtained from TargetScan Release 6.2 (http://www.targetscan.org) [31]. For each of the human and mouse essential genes having known paralogs, we made individual sets comprising the gene and all of its paralogs. We calculated the mean micro-RNA target sites of each of such sets for the two species. We considered the mean value of all sets within a species to obtain the mean micro-RNA target sites for that species.

### Evolutionary Rate

Evolutionary rates of the human and mouse genes were calculated as the ratio of nonsynonymous nucleotide substitution per nonsynonymous sites (dN) and synonymous nucleotide substitution per synonymous sites (dS), from the biomart interface of ensemble 71 (http://www.ensembl.org/biomart/martview) [29], using rat (*Rattus norvegicus*) as an outgroup. We obtained the dN and dS of human and mouse genes from their corresponding one-to-one rat orthologs. We compared the dN/dS ratios of nonredundant sets of human and mouse essential genes’ paralogs.

### Statistical Analyses

Statistical analyses of the entire work were performed using SPSS v.13 and in house PERL Script. Mann-Whitney U test was used in SPSS to compare the mean values of different variables between two classes of genes. We used our in house PERL Script to perform two-sample Z-test for comparing relative proportions of a variable between two gene groups.

## Results and Discussions

We compared the duplication of human and mouse essential genes and noticed that the tendency of essential genes to remain as duplicate copy varies between human and mouse. In human, the proportion of essential genes is higher among the duplicated subsets compared to the singleton genes; whereas in mouse, the reverse was observed. We observed that in mouse among 2098 singleton genes, 994 genes are essential (47.38%) and among 3771 duplicated genes, 1563 genes are essential (41.45%) [Z = 4.391, confidence level 99%; P<0.0001, two sample Z-test] whereas, in humans, among 7563 singleton genes, 486 genes exist as essential (6.43%) and among 10837 duplicated genes, 984 are essential (9.08%) [Z = −6.523, confidence level 99%; P<0.0001, two sample Z-test]. The overall proportion of essentiality is higher in mouse, which may be due to the fidelity of the methods applied to detect essential genes [27] or the unavailability of the complete essentiality data, but within species (where the same method is used to detect essentiality), gene essentiality should contribute equally among singletons and duplicates, which is however, not the case, as our observations indicate a higher probability of retaining the essential genes as duplicates in humans but not in mouse. A previous study reported that developmental genes are more essential than non-developmental ones [19] and their abundance may result higher essentiality for a particular gene group relative to other, which led us to hypothesise that the overrepresentation of developmental genes in a particular gene group may influence the overall trend. To explore if this is the case in our experiment, we discarded the developmental genes and calculated the proportion of essential genes in singleton and duplicate for human and mouse non-developmental genes only (see materials and methods for details). Here also, we obtained a similar trend (Table 1), which indicates that the results are not influenced by developmental genes. Therefore, we continued our study including both the developmental and nondevelopmental mouse and human genes.

**Table 1:** Proportion of essential genes among singleton and duplicates of mouse and human non-developmental genes.

Species	Gene group	Total genes	Essential genes	Proportion of essential genes	Z-score and P value
Mouse (*Mus musculus*)	Singleton	1237	462	37.348	Z = 5.0323 (Confidence level 99%) P <0.0001
	Duplicates	2301	669	29.074	
Human (*Homo sapiens*)	Singleton	6347	332	5.231	Z = −3.7168 (Confidence level 99%) P = 0.0002
	Duplicates	8581	575	6.701	

Another possible bias in our dataset may arise due to the age of the duplicates. Previous studies showed that the genes originated from old duplications are more likely to be essential than singletons [24]. Therefore, the age of genes have an influence in gene essentiality, which may lead to overestimation of human essential genes as duplicates in our dataset as we have considered duplicates as the genes having at least one paralogous copy, no matter how ancient it is. This bias was corrected by considering the phyletic age of the genes to calculate the overall proportion of essentiality [21] (see materials and methods) in singleton and duplicated mouse and human genes. We did not consider the duplication age (the origin of most recent duplication event) as our dataset also contains singletons and hence, phyletic age will be a more suitable measure. After correcting the age bias, we still obtained the same trend in proportion of essential genes in singletons and duplicates in both species (Table 2).

**Table 2:** Proportion of essential genes as weighted average among singleton and duplicates of mouse and human.

Species	Gene group	Total genes	Proportion of essential genes as weighted average (P_E_ = f_old_ × P_E_^old^ + f_young_ × P_E_^young^)	Z-score and P value
Mouse (*Mus musculus*)	Singleton	2098	47.379	Z = −4.392 (Confidence level 99%) P <0.0001
	Duplicate	3771	41.448	
Human (*Homo sapiens*)	Singleton	7563	6.426	Z = −6.535 (Confidence level 99%) P <0.0001
	Duplicate	10837	9.081	

Our study contradicted the previous study of Liao and Zhang [18] which entails that mouse singleton and duplicate genes have an equal proportion of essential genes. This may result from the difference in essential gene collection procedure followed in Mouse Genome Informatics (MGI) which they used and OGEE databases which we have used. However, our result of mouse genes essentiality is consistent with that shown by two more recent studies [21,24]. Thus, with no further controversy, we wanted to comprehend why essential genes prefer to remain as duplicates in humans. Firstly, we contemplated that human genes may be maintained to keep an extra copy for functional compensation. However, the higher connectivity (Hub like nature) of essential genes which was revealed in many previous studies [32–35] demands a stringent regulation, in order to maintain the whole protein interaction network dosage-balanced. Moreover, duplication leading to the increase in dosage may not be favourable and, as a result, duplicates must either be diversified [36] or kept silent (dosage-balanced) [16].

To investigate whether the diversification supports the fixation of duplicate copies of essential genes in the human genome, or the duplicates are maintained as a backup system under stringent dosage-regulatory mechanism, we compared the essential genes and their paralogs between mouse and humans.

**Fig 1:**
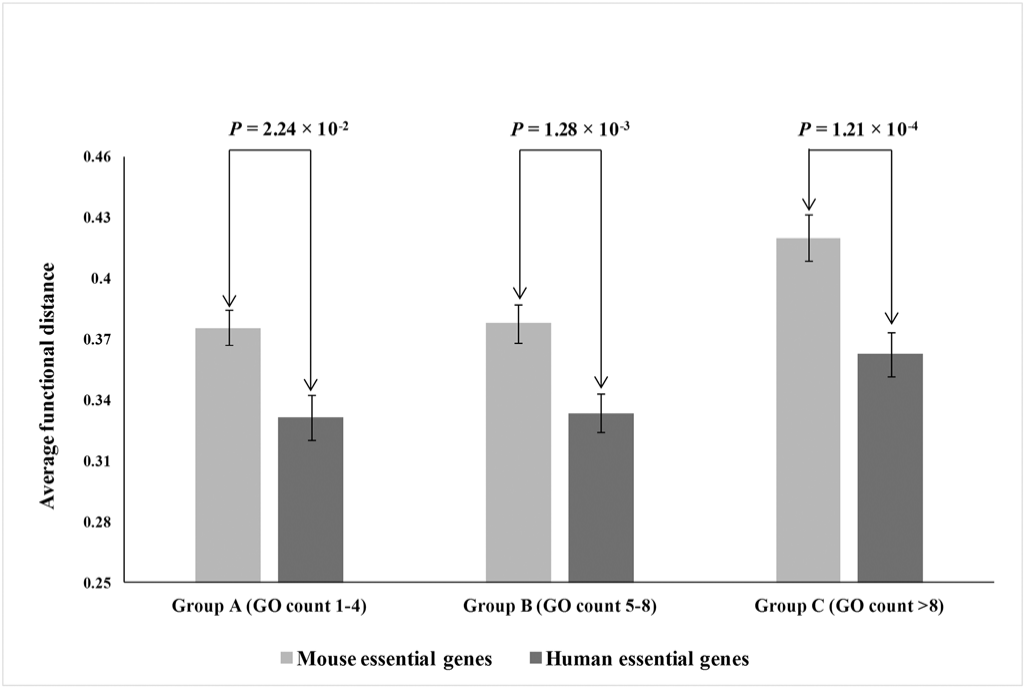
Average functional distance between mouse and human essential genes among three groups according to their count of GO terms. Group A with GO count 1–4, Group B with GO count 5–8 and Group C with GO count >8 (error bars indicate standard errors).

Firstly, we wanted to explore if the essential genes are duplicated for becoming functionally diversified and fixed in the genome. For this, we considered GO annotations for each human and mouse essential genes and their corresponding paralogous copies from Ensembl 71 biomart interface [29] for the GO domain Molecular function. Using the Czekanowski—Dice distance formula [30] (see materials and methods), we have obtained a significantly lower (P = 3.73×10^-6^, Mann-Whitney *U* test) functional distance value in human duplicated essential genes (Average functional distance = 0.340, N = 954) than in mouse duplicated essential genes (Average functional distance = 0.385, N = 1536). However, the Czekanowski—Dice distance formula we used here is sensitive to the number of go terms associated with a gene, which may vary from species to species. Therefore, for an unbiased cross-species comparison of functional distance, we binned our dataset into three groups containing according to their go id count (see materials and methods). We observed a significantly lower functional distance in human essential genes than the mouse counterparts in all three groups [Fig. 1], suggesting a tendency of retaining the human duplicated copies of essential genes *per se* as backup copies.

Although we observed that human essential duplicates are functionally less diverged than mouse, we were curious to understand the occurrence of pseudogenized paralogs among essential genes of both species. As our main dataset contains essential genes of human and mouse, no occurrence of pseudogene was observed. However, among the paralogs, we did not find any significant difference between mouse (0.82%) and human (0.50%)(Z = −1.584, P = 1.13×10^-1^, two sample Z-test), which may be due to the low proportion of pseudogene occurrence in both species (S3 Dataset). The low proportions of pseudogenes in our mouse and human essential genes’ paralogs are normal as we are considering paralogs of the genes with crucial functions. However, when we considered the proportion of paralog pseudogenization for each human and mouse essential duplicate genes having at least one pseudogenized paralog (see materials and methods), the proportion of paralog pseudogenization were found to be lower in human essential genes than in the mouse counterpart (Proportion of paralog pseudogenization in mouse = 0.178, Proportion of paralog pseudogenization in human = 0.048; P = 1.44×10^-7^, Mann-Whitney U test, N_mouse_ = 17, N_human_ = 63). This result suggests that mouse essential genes’ paralogs can become pseudogenized more easily. In other words, human essential genes retain their functionality more readily, which in turn can help them to serve as functional backup copies, as we have previously shown that they are functionally more similar to their ancestral genes.

The human essential genes in our study were observed to show lower functional divergence. Thus, we hypothesize that the essential gene duplicates are functionally redundant and they may be maintained as backup copies. However, the maintenance of newly synthesized duplicates is very crucial and often performed by micro-RNA mediated post-transcriptional regulation, which may give support to the backed up essential genes by reducing their expression [37]. Therefore, to measure the ability to maintain the backed up duplicates, we measured the average micro-RNA target sites for mouse and human essential genes and their duplicates (see materials and methods for details). Consistent with our expectation, we observed a significantly higher (*P* = 3.35×10^-6^; Mann-Whitney *U* test) micro-RNA target sites in duplicated essential genes of human (Mean micro-RNA count 19.15, Number of sets = 742) than in mouse (Mean micro-RNA count 15.82, Number of sets = 1202), suggesting the robust regulation by micro-RNAs after the duplication of essential genes enables humans to maintain the redundant copies.

**Fig 2:**
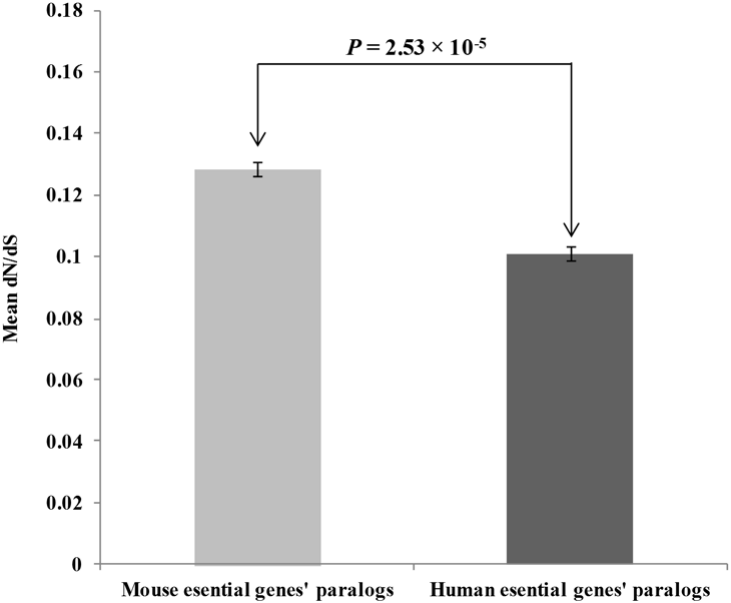
Mean dN/dS value of mouse and human essential genes’ paralogs (error bars indicate standard errors).

We observed the human essential duplicate genes mostly prefer to remain functionally redundant and can be maintained as backup copies, being able to escape the dosage imbalance. However, as the gene duplication is the mean of providing raw materials for genome evolution [4], we were interested in understanding the selection pressure on these backed up copies. Now, as the essential duplicates are functionally less divergent and dosage-balanced, their paralogs must be evolutionarily more conserved, in order to serve as backup copies upon future needs. We measured the evolutionary rates of human and mouse duplicated essential genes’ paralogs, in terms of the ratio of nonsynonymous substitution rates per nonsynonymous sites (dN) and synonymous substitution rates per synonymous sites (dS) [see materials and methods] and obtained a significantly lower evolutionary rate of human counterpart (dN/dS_human_ = 0.101, dN/dS_mouse_ = 0.128, P = 2.53×10^-5^, Mann Whitney U test, N_mouse_ = 2931, N_human_ = 1651), indicated by their lower dN/dS ratio [Fig. 2]. This indicates that the redundant copies of human essential duplicates are evolutionarily conserved and may serve as backup copies upon future requirement, having the potential to increase the gene deletion fitness effect.

## Conclusion

Gene duplication generates multiple copies of a gene that are initially functionally redundant, and their retention demands either functional diversification or regulation of the protein dosage. In this study we showed that human essential genes are mostly retained as duplicates, a trend which is different from mouse, with the duplicated copies being functionally more redundant in humans. Consistent with this, the evolutionary rate of these redundant human paralogs of essential genes is lower than that in mouse. We showed that these redundant human duplicates can be maintained due to the presence of more efficient dosage-regulation. Our study sheds light on the importance of the backup copies to restore the fitness effect of gene deletion, thereby increasing the fitness in humans. This study opens the future direction for in depth analysis of duplicated essential genes and their role in the human protein evolution.

## Supporting Information

S1 Dataset Mouse and Human genes used in this study. This dataset contains the essentiality, duplicability, involvement in development and phyletic age data of mouse and human genes.
(XLSX)

S2 Dataset The duplicated pairs of Mouse and Human genes. This dataset contains the duplicate pairs for mouse and human genes used for functional distance measurement.
(XLSX)

S3 Dataset The pseudogenization status of the paralogs of Mouse and Human essential duplicate genes. This dataset contains the pseudogene annotation for all mouse and human genes under study.
(XLSX)
